# Machine learning predictive modelling for identification of predictors of acute respiratory infection and diarrhoea in Uganda’s rural and urban settings

**DOI:** 10.1371/journal.pgph.0000430

**Published:** 2022-05-11

**Authors:** Rornald Muhumuza Kananura

**Affiliations:** 1 London School of Economics and Political Science, Department of International Development, London, United Kingdom; 2 Makerere University School of Public Health, Department of Health Policy Planning and Management, Kampala, Uganda; University of California San Francisco, UNITED STATES

## Abstract

Despite the widely known preventive interventions, the dyad of acute respiratory infections (ARI) and diarrhoea remain among the top global causes of mortality in under– 5 years. Studies on child morbidity have enormously applied “traditional” statistical techniques that have limitations in handling high dimension data, which leads to the exclusion of some variables. Machine Learning (ML) models appear to perform better on high dimension data (dataset with the number of features p (usually correlated) larger than the number of observations N). Using Uganda’s 2006–2016 DHS pooled data on children aged 6–59 months, I applied ML techniques to identify rural-urban differentials in the predictors of child’s diarrhoea and ARI. I also used ML to identify other omitted variables in the current child morbidity frameworks. The predictors were grouped into four categories: child characteristics, maternal characteristics, household characteristics and immunisation. I used 90% of the datasets as a training sets (dataset used to fit (train) a prediction model), which were tested or validated (dataset (pseudo new) used for evaluating the performance of the model on a new dataset) on 10% and 30% datasets. The measure of prediction was based on a 10-fold cross-validation (resampling technique). The gradient-boosted machine (ML technique) was the best-selected model for the identification of the predictors of ARI (Accuracy: 100% -rural and 100%-urban) and diarrhoea (Accuracy: 70%-rural and 100%-urban). These factors relate to the household’s structure and composition, which is characterised by poor hygiene and sanitation and poor household environments that make children more suspectable of developing these diseases; maternal socio-economic factors such as education, occupation, and fertility (birth order); individual risk factors such as child age, birth weight and nutritional status; and protective interventions (immunisation). The study findings confirm the notion that ARI and diarrhoea risk factors overlap. The results highlight the need for a holistic approach with multisectoral emphasis in addressing the occurrence of ARI and diarrhoea among children. In particular, the results provide an insight into the importance of implementing interventions that are responsive to the unique structure and composition of the household. Finally, alongside traditional models, machine learning could be applied in generating research hypotheses and providing insight into the selection of key variables that should be considered in the model.

## Introduction

The global inequities in child mortality have consistently remained large, with sub-Saharan Africa contributing the largest share [1–3]. In 2019, the under-five mortality rate in sub-Saharan Africa was 78 per 1000 live births, twice higher than the global rate and at least 16 times higher than high-income countries’ average [2]. Despite the widely known yet cost-effective preventive and protective measures [4], pneumonia and diarrhoea have persistently appeared among the leading causes of under-five mortality. Globally, pneumonia and diarrhoea have recurrently contributed an estimate of at least 24% of all under-five years mortality causes [1, 3, 5, 6] and the burden of the two diseases remains high in sub-Saharan Africa [7–9].

In Uganda, the prevalence of *suspected pneumonia* (hereinafter referred to as “Acute Respiratory Infection (ARI)”) and diarrhoea in the recent Uganda Demographic and Health Survey (UDHS) is estimated at 20% and 9%, respectively [10]–making Uganda among the top 5 countries with a high proportion of children that experience diarrhoea and ARI (S1 Fig). Like other sub-Saharan African countries, pneumonia and diarrhoea are the leading causes that burden the health facilities in Uganda. For instance, in Uganda, out of the 8.8 million under-five health facility outpatient admissions in 2017/2018, 13.3% were due to either pneumonia (4.3%) or diarrhoea (9.1%) [11]. During the same period, out of 0.68 million under-five health facility inpatient admissions, 22% were due to pneumonia (14%) and diarrhoea (8.1%) [11]. The recurrent episodes of pneumonia and diarrhoea among children in sub-Saharan Africa lead to not only persistent high child mortality and long-term disabilities in the region but also catastrophic expenditure as well as a long-term economic burden on the individuals and families [12–16]. For instance, some of the studies on the cost of diarrhoea and pneumonia done in Ethiopia and Uganda indicated an average of $62–64 for each hospitalised episode of pneumonia and an average of $79 for each hospitalised episode of diarrhoea [12, 14].

Diarrhoea and ARI have common risk factors, including poor nutrition, poor hygiene and sanitation, and poor living conditions [4, 17]. Additionally, both diseases lead to other health consequences such as anaemia that could later increase the probability of death or inhibit children from thriving. Noteworthy, the association of the risk factors with the prevalence of morbidities is a complex process of interrelated mechanisms [7]. The health sector cannot solely address such complexity. Indeed, the sustainable development goals (SDGs) framework and the Global Strategy for Women’s, Children’s and Adolescents’ Health underscore the interrelations between most of the goals by highlighting how progress in one area may affect progress in many others [18, 19]. Such emphasis calls for a multisectoral approach to designing and implementing child health interventions [18, 19].

Ending the preventable causes of child death, such as pneumonia and diarrhoea, is among the global health priorities [18, 19]. The sustainable development goals (SDG frameworks) include interrelated goals that contribute to reducing deaths due to preventable causes. To improve children’s health and well-being, the Global Strategy for Women’s, Children’s and Adolescents’ Health recognises a range of health-related goals and targets that must be addressed through a multisectoral approach [20]. Some of the SDG indicators that are relevant in reducing the occurrence of pneumonia and diarrhoea include 1) SDG target 3.9 –reducing mortality and morbidity due to water and soil pollution and contamination; 2) SDG target 2.2 –ending all forms of malnutrition: child stunting, child wasting, child overweight; 3) SDG target 6.1 –achieving universal and equitable access to safe and affordable drinking water for all; 4) SDG 6.2 –achieving access to adequate and equitable sanitation and hygiene for all; 5) SDG 7.1 –ensuring universal access to affordable, reliable and modern energy services; 6) SDG 11.1 –ensuring access for all to adequate, safe and affordable housing and basic services and upgrade slums. And end open defecation, paying attention to the needs of women and girls and those in vulnerable situations; and 7) including other parts of targets in goals on poverty, hunger, education, gender.

Furthermore, children’s health and well-being depend dramatically on where they are born or reside [21]. Such differences could be due to the residential variations in the earlier alluded risk factors: environmental, healthcare access, socio-economic and demographic characteristics, and food access. For instance, rural dwellers’ children are usually susceptible to a high risk of diseases such as malaria, pneumonia, and diarrhoea [22, 23]. The high risk of morbidity could be explained by poor environmental measures such as lack of access to clean water, poor toilet coverage, indoor pollution and socio-economic factors such as poverty and low levels of education [24]. However, even the urban marginalised dwellers are susceptible to a high risk of morbidities such as malaria, pneumonia and diarrhoea [22, 23, 25]. The urban marginalised are more characterised by risk factors that have been mentioned to increase the likelihood of morbidities prevalence among rural dwellers [22]. Nonetheless, information on the differences in the predictors of pneumonia and diarrhoea between urban and rural settings is elusive since the analysis usually considers rural-urban disaggregation, which masks the vulnerable groups living in urban areas.

In light of the above, there is a need for risk assessment approaches that can comprehensively provide a set of variables that may describe children at risk of contracting morbidities and how these variables may differ across the place of residence. Such assessment is crucial in setting priority areas of focus and steering the collaboration and integrations at different societal levels, and ultimately addressing the fragmentation of various pieces of interventions. For example, we know that pneumonia and diarrhoea have numerous risk factors and determinants that are usually correlated, limiting the number of variables in the traditional models such as logistic regression, linear regression, and Cox regression. On the one hand, because of the limitations of traditional statistical approaches in handling highly dimensional and correlated variables (collinearity assumption), variables are usually dropped out of the model. Normally, the exclusion or inclusion of variables depends on the researchers’ interests. On the other hand, dimensionality reduction approaches such as factors analysis or principal component analysis are always applied to create indices, thus leading to the loss of information.

Furthermore, the conclusion and interpretation of traditional models are based on statistical significance (p-value), where the focus is usually on variables with a lower p-value for a given level of significance. Conclusion and interpretation of data based on p-value s may provide limited information about the data [26]. Additionally, large samples generate smaller p-values and therefore relying on p-values may lead to claim support for results [27]. Drawing on the limitations of the traditional models that have been applied in the current literature, the available frameworks or theories and conclusions based on their formulation of traditional analysis approaches may lead to inappropriate decisions and fragmentation of interventions [28, 29].

Building on the traditional statistical models, such as linear and non-linear regression models applied in the current literature, I examine the urban-rural differences in the predictors of pneumonia and diarrhoea using Machine Learning (ML) approaches. The analysis was applied to a pooled dataset of 2006–2016 UDHS. ML models such as lasso, random forest and deep learning appear to perform better than the traditional linear and non-linear models on high dimensional datasets or correlated variables and datasets with more variables than observations [29, 30]. The ML modelling idea lets the algorithm determine how the outcome and independent variables are linked [29, 31]. So far, ML has not been extensively applied to the available cross-sectional data in LMICs. The key applications refer to clinical research data [32–38] and only a handful studies have applied ML using cross-sectional population health data [28, 39]. Thus, to my knowledge, this study is the first to apply an algorithmic modelling approach to identify predictors of pneumonia and diarrhoea in LMICs settings based on cross-sectional surveys. I study how the predictors of ARI and diarrhoea vary across the places of residence (rural versus urban). The identification of these predictors not only provides a set of measures for vulnerable children at risk of ARI and diarrhoea but also provides a new direction for rethinking the implementation mechanism of preventive interventions that target communities, families, and children with such identified characteristics. Finally, studying how the predictors by place of residence differ may give us an insight into an area-specific intervention package.

### Analysis and theoretical approaches to the measures of child morbidity risk factors

While available studies [40–43] have based their analysis on Mosley and Chen model to understand the determinants of child morbidities in developing countries [44], the application of traditional analysis approaches appear to have limited the consideration of other important variables. The framework by Mosley and Chen considers a range of social, economic, cultural, and health system variables that impact child health and survival through a set of proximate determinants. These are categorised as maternal demographics and socio-economic, environmental, nutrient deficiency, and geographic position. The occurrence of diarrhoea has been indicated to be highly associated with underweight or malnourished children [45–48]. The socio-economic factors associated with ARI and diarrhoea among children include the wealth position of the family, parents’ education level and employment status [42, 43]. For instance, in their study on morbidity among children in Bangladesh, Kamal et al.,2015 indicate that children from low-income families were at least 40% more likely to suffer from a common illness than those from less low-income families [43]. The demographic factors associated with ARI and diarrhoea among children include maternal age, marital status, and household head age [41, 42]. The main environmental factors that have been identified to increase morbidity among children are poor sanitation and hygiene, access to unsafe water, and household air pollution [43]. Notably, and like Hill, 2003 indicates, birth weight as a potential risk factor for child’s health throughout their childhood age is missing in the Mosley and Chen framework [49], although this could be correlated with the nutrition deficiency that is indicated in the framework. The association of LBW with diarrhoea and ARI has been revealed in some studies conducted in low and middle-income countries [48, 50–52].

While a sheer volume of research has offered a lot in understanding and improving child health, building on this available evidence, there is a need to advance research methodologies that may contribute to the generation of new interventions and implementation approaches in this area. Our current methods in this field majorly rely on “traditional” statistical analysis approaches that are based on several assumptions. For instance, the assumption of collinearity and the number of parameters versus sample size may lead to the exclusion of important variables, leading to elusive conclusions. Reducing dimensionality or exclusion of parameters reduces the information of model accuracy prediction [29].

Furthermore, what has been missing is that the child health and well-being factors may vary across and within the countries/communities [53, 54], and perhaps the magnitude of each factor may vary. As alluded to earlier, the places of residence are characterised differently by community behaviours such as myths and geographic characteristics such as exposure to environmental risk factors that contribute to the high incidence and re-occurrence of morbidities.

Contrary to the different approaches that previous studies have adopted in the same field, which is a key strength of this study, I use an algorithmic approach to identify the potential rural-urban differentials in the predictors of diarrhoea and ARI among children in Uganda. I compare the ML results with the traditional logistic regression to determine the model with better accuracy in predicting ARI and diarrhoea. Instead of generating indices that have been used to generate some measures, including wealth index, I include all the variables available in the dataset that are known to affect the health and well-being of children to generate diarrhoea and ARI predictive algorithm. Notably, the application of different approaches and modelling in data analysis is usually affected by the availability of the relevant data, which also affects ML approaches. Therefore, I am not claiming that ML would be the best alternative and unique approach that would not suffer from data availability limitations.

## Methods

### Data source

To have a sufficient dataset for identifying predictors of this study’s outcomes, I pooled the UDHS data collected between 2006 and 2016 and publicly accessed as of March 2020. For all waves of data collection considered under this study, the DHS used a multistage, stratified sampling design with households as the sampling unit. DHS has a standard household questionnaire that collects information on characteristics of the household’s dwelling unit, such as water sources, type of toilet facilities, materials used for the floor of the dwelling unit, and ownership of various household items. The household tool also includes a household roster that captures data on each household member’s age and sex, which is used to identify women, men, and children eligible for individual interviews, anthropometry measurement, and anaemia testing. Furthermore, the DHS uses the women’s questionnaire to collect information related to their reproduction and fertility history, sexuality, pregnancy and birth experience, and child health. For example, the questions on child health include asking eligible women to recall if their children had symptoms of diarrhoea and ARI in the recent period preceding the day of data collection. Information on vaccination coverage is collected from the child’s health card or the mother’s direct report. In Uganda, the pneumococcal conjugate vaccine was introduced in April 2013. Although the rotavirus vaccine became part of the national routine immunisation schedule in February 2018, while it was not part of the schedule at the time of the recent (2016) survey, some private health facilities were offering the rotavirus vaccine for a fee.

Furthermore, questions that are used to identify a child who experienced a recent episode of diarrhoea and ARI are 1) Has (NAME) had diarrhoea in the last two weeks?; 2) Has (Name) had an illness with a cough at any time in the last two weeks?; 3) When (NAME) had an illness with a cough, did she breathe faster than usual with short, rapid breaths or have difficulty breathing?; and 4) Was the fast or difficult breathing due to a problem in the chest or to a blocked or runny nose?. These questions were the same for all data collection periods (2006, 2011, 2016) considered in this study.

The data were pooled using IPUMS-DHS online system [55], by choosing children under the age of five years and their related records. This included only children aged 0–59 months born in the five years preceding the survey. S1 Table summarises the data variable for each of the categories that were selected using IPUMS-DHS.

### Response and explanatory variables

The key outcome (response) variables are the prevalence of ARI and diarrhoea. In the DHS, the measurement of ARI is based on the occurrence of short, rapid breathing that is chest-related and/or difficult breathing that is chest-related that is based on women’s ability to recall. This was categorised as ‘1’ presence of disease and ‘0’ otherwise. For diarrhoea, women are asked to recall if their children have had diarrhoea in the last 2 weeks, categorised as ‘1’ presence of disease and ‘0’ otherwise.

The key predictors (explanatory) were selected from a set of 42 variables that were categorised as mothers’ demographic position, household assets, household environmental characteristics, and child characteristics. The consideration of the covariates was based on a literature review. Under household environmental characteristics, studies have indicated how the number of people living under one roof (congestion), the house structure, availability of toilets, treatment of drinking water and indoor air pollution (cooking inside the house or use of firewood) are associated with the occurrence of pneumonia and diarrhoea [42, 43, 56–58]. For child characteristics, studies have indicated how the birth weight of the child, birth category (multiple or single), child age, child sex, and anthropometric measures (height and weight) are associated with the occurrence of suspected pneumonia and diarrhoea [48, 50–52, 58, 59]. Furthermore, studies have also indicated how the mothers’ demographic position measures such as maternal age, household head age, household head sex and maternal education are associated with the occurrence of suspected pneumonia and diarrhoea [56, 58, 60]. Lastly, households’ assets that have often been used as the measure of household wealth through principal component analysis have been indicated to be associated with pneumonia and diarrhoea [56, 57, 60]. S1 and S2 Tables summarise the data variable for each of the categories that were selected using IPUMS-DHS.

### Data analysis

Data cleaning and descriptive statistics were done in Stata 16. ML analysis was performed using R statistical software version 3.6.1 with the caret package [61]. During data cleaning, I excluded 7% of children who were not staying with the mothers on the day of the interview, which is not in line with the standard DHS analysis [62] and may lead to differences in the reported estimates of ARI and diarrhoea. The premise was that including children that were not living with their parents at the time of data collection may lead to underestimation as mothers may not be sure of their health status in recent days when they were not home. Therefore, the total sample size for the pooled dataset includes only children who were residing with their parents at the time of the interview is 27687. Table 1 shows the distribution of unweighted sample size across years of data collection.

**Table 1:** Distribution of unweighted sample size across years of data collection.

	Sample size of all children				Sample size that only includes children residing with their parents at the time of the interview			
Place of residence	2006	2011	2016	Total sample	2006	2011	2016	Total sample
	**0–59 months**							
Rural	6,746 (88.9%)	5,772 (78.4%)	12,037 (81.8%)	24,555 (82.8%)	6,436 (89.4%)	5,492 (79.6%)	11,222 (82.5%)	23,150 (83.6%)
Urban	847 (11.1%)	1,583 (21.6%)	2,673 (18.2%)	5,103 (17.2%)	763 (10.6%)	1,407 (20.4%)	2,367 (17.5%)	4,537 (16.4%)
Total	7,593	7,355	14,710	29,658	7,199	6,899	13,589	27,687
	**6–59 months**							
Rural	6035 (89%)	5171 (78.9%)	10824 (81.6%)	22030 (82.7%)	5726 (89.6%)	4894 (80.1%)	10023 (82.4%)	20643 (83.7%)
Urban	747 (11%)	1387 (21.2%)	2445 (18.4%)	4579 (17.2%)	664 (10.4%)	1213 (19.9%)	2142 (17.6%)	4019 (16.3%)
Total	6782	6558	13269	26609	6390	6107	12165	24662

The dataset was filtered based on the place of residence to generate two datasets: Urban and Rural residents’ datasets. Modelling was done on each of the datasets separately. To identify the community and household predictors of ARI and diarrhoea, I used two models based on ML techniques and a traditional logistic regression model to select the best model that predicts the probability of ARI and diarrhoea prevalence. The ML models were lasso logistic and gradient boosting machine. Based on the theoretical framework, including available literature [42–44], variables (S1 Table) that could be associated with the occurrence of diarrhoea and ARI were entered in all the ML models. Instead of generating a latent variable or an index that is usually done through principal component or factor analysis, for instance, an assent index (a measure of wealth) that is based on the list of household assets; and an environmental index that is based on the household environmental characteristics, all the variables (S1 Table) were included in the models to determine those that were substantial in predicting the occurrence of diarrhoea and ARI.

**Fig 1:**
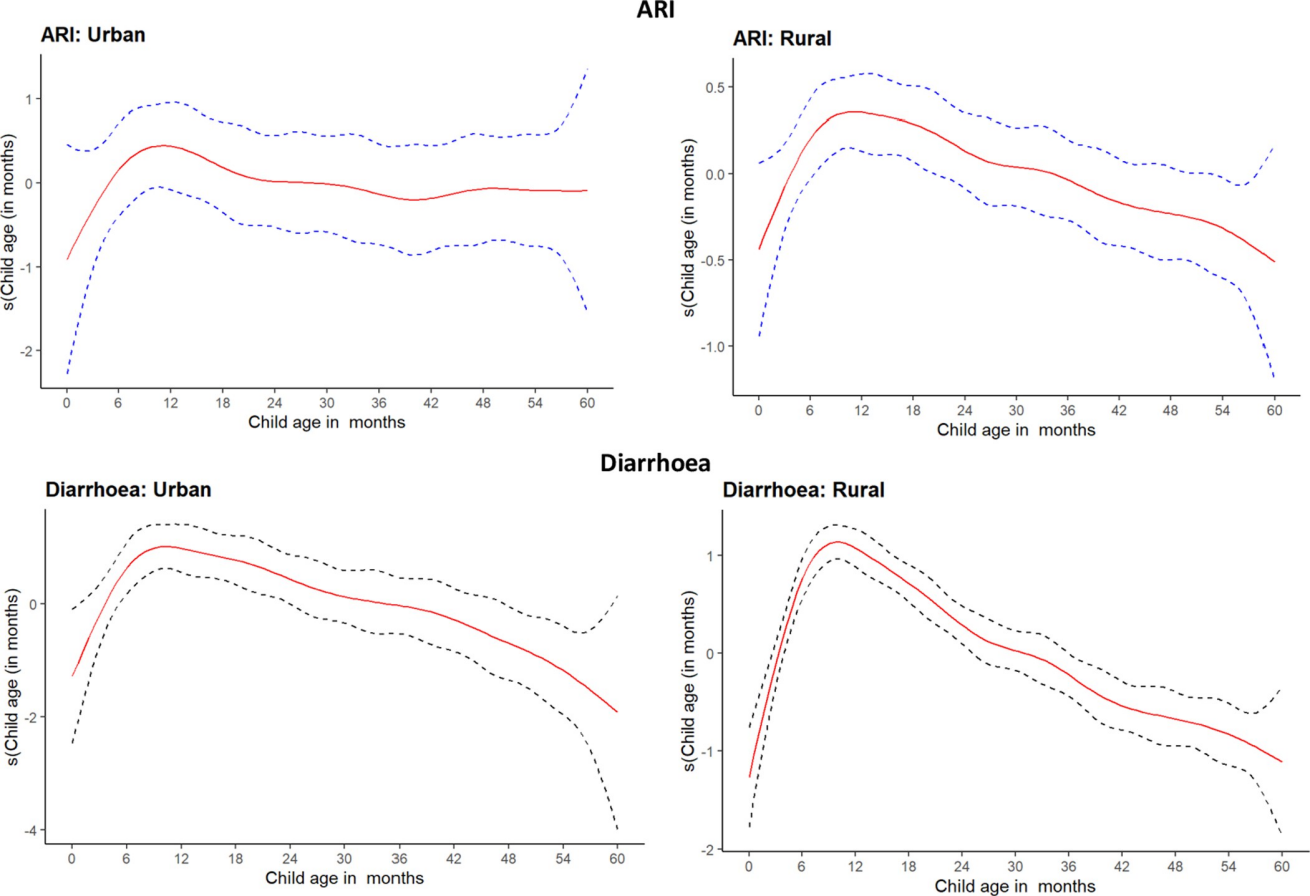
Assessment of the association of ARI and diarrhoea with children’s age.

Furthermore, in Uganda, some of the vaccines (rota-virus and pneumococcal vaccine) included in this manuscript are administered to children at the age of 6 months. As such, the modelling considered only children aged 6–59 months. However, before the modelling, I first assessed the association of diarrhoea and ARI with child age, which showed a steep curve in diarrhoea and pneumonia at 6 –indicating how quickly the child’s health can worsen within this period (Fig 1).

### Logistic regression

For each of the outcomes and place of residence, I ran the logistic regression model with all the independent variables included (S1 Table). The modelling was done on the training sets (90% of the dataset) and the 10% and 30% of the testing or validation sets. Both stepwise and backwards were considered in the selection of important variables.

### Lasso regularisation

When faced with many predictors (p), Lasso regularisation–an extended standard regression model- is superior in selecting important predictors (feature selection) that are more interpretable and more useful than the standard logistic regression. Unlike the standard logistic regression model, the lasso approach shrinks logistics regression coefficients towards zero, thereby performing variable selection [63]. Shrinking of coefficients helps to reduce the model complexity and multi-collinearity. It may be hard to select the model’s important variables with high dimensional data; however, through shrinkage, parameters with low coefficients are shrunk to zero, which reduces the number of parameters. Reducing the number of parameters reduces variance and increases the bias (variance-bias trade-off). Bias is measured as the difference between the mean predicted values and the mean expected values, and the larger the difference, the higher the bias. Therefore, increasing bias may affect the model to accurately account for the relationship between the features and target of the data, generating inaccurate predictions. In this study, I performed Lasso with binomial link function using the *glmnet* package [63] implemented in R.

### Decision tree classification

The decision tree classifications are tree bagging, Random Forest (RF), and Gradient-Boosted Machine (GBM) [63]. Bagging as a technique of aggregating bootstraps [63] is used to reduce the variance in the decision tree predictions. It is done by combining the result of multiple classifiers modelled on different random sub-samples of the same training dataset [63] from which a separate prediction model for each sub-training set is generated, and later the overall mean prediction is generated. However, RF provides an improvement over the bagged tree by decorrelating the trees [63] and generating better predictions of the response variable by recursively splitting the data into more homogenous units (nodes) independent of the other. Furthermore, GBM is another ensemble approach applied to improve the predictions of the decision tree. Like the RF, boosting involves creating multiple copies of the original training dataset using the bootstrap, fitting a separate decision tree to each copy, dependent on each other, unlike the RF. Each tree is built on a bootstrapped dataset, dependent on the other trees, but in boosting, the trees are grown sequentially–each tree is grown using information from previously grown trees [63]. A mean prediction is later generated by combining all the trees [63]. Therefore, GBM is usually considered the best ML model approach [63]. In this study, GBM was the decision tree classification ML model that I applied. To get the best results, the model was tuned with the number of trees (500, 1000, 1500, and 2000) in an interval of 500, hyperparameter (1, 2, 3, 4, 5) and the training rate (shrinkage) (0.1, 0.01, 0.001) and the tuning parameters selected best on the combination of parameters that provider better accuracy.

For each area of residence (urban and rural), their respective datasets were split to 90% as training sets and the rest 10% as the testing sets. Modelling was done on the training sets (90%) and validated on testing sets (10%) and on a random sample of 30% observations (n) of the unsplit datasets. Note that the training set is used in running the model(s), while the testing set is used for validating the performance of the model(s) in other new datasets with the same parameters [64]. For both models, the measure of prediction was based on 10-fold cross-validation. After running the best model for selecting the most important variables, predictive probabilities were generated to assess the actual direction of the selected variables in affecting their respective outcomes.

### Model evaluation

Four measures were used in the selection of the best model: sensitivity, specificity, accuracy, and area under the roc curve.

#### Sensitivity

The sensitivity of a test is its ability to determine the patient cases correctly [65]. Sensitivity is the proportion of real positive cases that were predicted as positive. This implies that there could be another proportion of real positive cases that could be predicted as negative (false negative). This can also be presented in the form of a false negative rate (Eq 1).


$$\frac{True\;Positives}{True\;Positives+False\;Negative}$$ (1)

#### Specificity

The specificity of a test is its ability to determine the healthy cases correctly [65]. It is the proportion of real negative cases that were predicted as negative. This implies that there could be another proportion of real negative cases that could be predicted as positive (false positives). This can also be presented in the form of a false positive rate (Eq 2).


$$\frac{True\;Negative}{True\;Negative+False\;Positive}$$ (2)

#### Accuracy

The accuracy of a test is its ability to differentiate the patient and healthy cases correctly [65]. It is calculated as the proportion of true positive and true negative in all evaluated cases (Eq 3).


$$\frac{True\;Positives+True\;Negative}{True\;Positives+False\;Negative+True\;Negative+False\;Positive}$$ (3)

#### The area under the roc curve

I measured the prediction performance of each model by computing the ROC and the model with the highest ROC curve and accuracy estimates was selected.

#### Identification of the influential variables

The variable importance for the best model was also computed relative to the highest. The variable importance is a scaled measure with a maximum value of 100.

### Ethics

The datasets used in this study are publicly available in the DHS repository, with all identifier information removed. Thus, no ethics approval was required. However, permission was granted to download and use the datasets by the Demographic and Health Surveys (DHS) Program data archivist.

## Results

### Descriptive statistics

#### Maternal and household characteristics

As of 2016, the mean age of the children’s mothers and household heads was 27 ± 7 and 37 ± 12, respectively, which were not different from the 2006 and 2011 estimates. Almost 90% and 28% of the children’s caretakers or mothers had at least a primary and at least a secondary level of education, respectively, which increased by two times between 2006–2016. Regarding the household structure, as of 2016, 32%, 68% and 9% of the households had incomplete roofs, floors, and walls, respectively. During the same period, 36% of the household had shared toilets, 99% used wood or charcoal (22% for charcoal). S2 Table presents the maternal and household characteristics.

#### Child-related individual characteristics

S2 Table presents the child-related individual characteristics. As of 2016, 34% of the children were of 5^th^ and above birth position, and 3% were born as multiple, and the estimates were the same in the 2006 and 2011 reporting years. Within the same reporting year, 28% were stunted–declining by 9% between 2006–2016, 10% underweight for age (underweight)–reducing by 6% between 2006–2016, and 4% under-weight for height (wasted)–decreasing by 2% between 2006–2016. During the same reporting year, the average birth weight was 3.3 kg with a standard deviation of ± 0.8.

#### Trends in the prevalence of ARI and diarrhoea among under-5 of age children

Table 2 shows the prevalence of diarrhoea and ARI changes across time with their respective confidence intervals disaggregated by the place of residence. Overall, between 2006–2016, the national prevalence of suspected diarrhoea reduced by 6% point (27% in 2006 to 21% in 2016), while ARI reduced by 5% point. Disaggregating the estimates by place of residence, the prevalence of diarrhoea declined by 7% point in the rural area and 2% point in the urban area. Similarly, the prevalence of ARI reduced by 6% point in the rural area and 4% point in the urban area.

**Table 2:** Trends in the prevalence of ARI and diarrhoea among under-5 of age children.

*Category*	*Year*	*Diarrhoea*		*ARI*	
		*%*	*95% CI*	*%*	*95% CI*
Rural	2006	27.6	25.9–29.3	15.5	14.1–17.2
	2011	24.6	22.8–26.6	15.7	14.2–17.3
	2016	21.1	19.8–22.4	10.3	9.5–11.3
Urban	2006	21.3	17.3–25.9	11.8	8.3–16.5
	2011	23.7	20.0–27.9	13.8	11.1–17.1
	2016	18.6	16.6–20.7	7.6	6.1–9.5
National	2006	26.9	25.3–28.5	15.1	13.8–16.6
	2011	24.5	22.8–26.3	15.5	14.1–16.9
	2016	20.6	19.5–21.7	9.8	9.0–10.6

Note

1. CI represents the confidence interval.

2. The estimates are based on the live children who were staying with the parents or caretakers at the time of the interview.

### Rural-urban differences in the predictors of diarrhoea and ARI using 2006–2016 UDHS data

In the following sub-sections, I present the results on the predictors of diarrhoea and ARI that were identified using the ML modelling technique. I first present the results of the model assessment from which the best model was selected, and subsequently, present the key predictors that were identified based on the best model. Results on how the identified predictors affect the occurrence of morbidities are presented based on predicted probabilities.

#### Assessing the best model for selection

The best model was chosen based on the accuracy and sensitivity of the model in predicting the occurrence of ARI and diarrhoea (Table 3). Compared to the traditional logistic model and lasso logistic model, the GBM model was found to be superior in determining the potential predictors of ARI and diarrhoea. Contrary to other models, the GBM accuracy, sensitivity, specificity, and area under the ROC curve result in predicting the occurrence of ARI and diarrhoea among urban and rural residents were consistent (Table 3). While the traditional logistic model showed good accuracy in predicting ARI and diarrhoea, the likelihood of the model in identifying the actual cases (sensitivity) and false cases (specificity) was low and inconsistent across the datasets (training and testing datasets) (Table 3). Therefore, the interpretation of the results and conclusions are based on GBM predictions.

**Table 3:** Comparing the performance of logistic, Lasso and GBM models in the identification of the predictors of ARI and diarrhea.

	Rural			Urban		
	Testing set (10%)	Testing set (30%)	Training set (90%)	Testing set (10%)	Testing set (30%)	Training set (90%)
	**ARI**					
	**GBM**					
**Sensitivity**	1.00	1.00	1.00	1.00	1.00	1.00
**Specificity**	0.90	0.91	0.90	0.96	0.97	0.96
**Accuracy**	0.91	0.91	0.91	0.96	0.97	0.96
**ROC**	0.95	0.94	0.94	1.00	1.00	1.00
	**Lasso**					
**Sensitivity**	0.33	0.87	0.86	0.75	0.75	0.75
**Specificity**	0.87	1.00	0.43	0.93	0.94	0.94
**Accuracy**	0.86	0.87	0.87	0.93	0.93	0.93
**ROC**	0.73	0.75	0.75	0.91	0.88	0.90
	**Logistic**					
**Sensitivity**	0.08	0.42	0.04	0.59	1.00	0.38
**Specificity**	0.99	0.98	1.00	0.97	1.00	0.99
**Accuracy**	0.87	0.91	0.87	0.94	1.00	0.93
**ROC**	**0.78**	**0.90**	**0.75**	0.97	1.00	**0.89**
**Suspected diarrhoea**						
	**GBM**					
**Sensitivity**	0.69	0.73	0.70	1.00	1.00	1.00
**Specificity**	0.78	0.77	0.78	1.00	1.00	1.00
**Accuracy**	0.77	0.77	0.77	1.00	1.00	1.00
**ROC**	0.80	0.76	0.78	1.00	1.00	1.00
	**Lasso**					
**Sensitivity**	NA**	NA**	NA**	NA**	NA**	NA**
**Specificity**	0.74	0.75	0.75	0.78	0.79	0.78
**Accuracy**	0.74	0.75	0.75	0.78	0.79	0.78
**ROC**	0.71	0.68	0.70	0.74	0.76	0.74
	**Logistic**					
**Sensitivity**	0.28	0.43	0.18	0.46	1.00	0.38
**Specificity**	0.94	0.89	0.96	0.96	1.00	0.96
**Accuracy**	0.77	0.78	0.76	0.85	1.00	0.83
**ROC**	**0.77**	**0.84**	**0.74**	0.86	1.00	**0.81**

** The predictive True Positive was zero.

### Key household and individual predictors of diarrhoea in rural and urban areas

Table 4 presents the differences in identified predictors of diarrhoea in traditional logistic regression and GBM. In rural areas, using the traditional logistic regression model based on 95% statistical significance, six variables (child age, childbirth order, maternal age, safe drinking water, and pneumococcal vaccine) would be considered the most important predictors of diarrhoea (Table 4). However, the GBM identified 17 important predictors (Table 4). While in urban areas, using the traditional logistic regression model based on 95% statistical significance, 10 variables would be considered the most important predictors of diarrhoea and 13 were identified under GBM (Table 4).

**Table 4:** Differences in identified predictors of diarrhoea in traditional logistic regression and GBM.

	Rural		Urban	
	Logistic regression	GBM	Logistic regression	GBM
Variables	P-value	Level of importance	P-value	Level of importance
**Child characteristics**				
Child age	0.04	100	0.006	100
Birth weight	-	26.6	-	16.9
Birth order	0.008	3.3		10.1
Nutritional status: Stunted			-	9
**Maternal characteristics**				
Maternal age	0.019	28.1	0.031	16.4
Maternal occupation: Professional	0.105			
Maternal occupation: Agriculture	0.146		0.041	-
Maternal occupation: Domestic and services	0.011			
Maternal occupation: Manual worker	0.094	13.2		
Education level: Primary	0.037		0.044	-
Maternal education: Secondary	0.015			
Education level: Higher level	-	-	-	6.9
**Household characteristics**				
*Hygiene and sanitation*				
Toilet Type: Unimproved	-	20.8	0.161	9.5
Number of households that share toilet	-	15.8		
Household treats water for drinking	0.016			
*Household asset position*				
Household has electricity	-	3.3	0.003	10.4
Asset ownership: Radio		3.8		
Household asset: TV	0.986		0.027	-
*Household congestion measure*				
Number of household members		6.3	0.017	-
Number of household members per room	0.102	-	0.001	21.6
Number of old people in the household	-	7	0.002	27.1
Number of under-5 children			0.007	6.3
*Household head characteristics*				
Age of household head	-	26.5	-	9.5
Household head sex: Male	0.098	7.2		
*Exposure to smoke*				
Cooking place: Separate room or house	-	5.3		
Cooking fuel: Charcoal	-	4.9		
*Household structure*				
Incomplete floor	0.053	-	-	6.6
**Child vaccination**				
vaccine: 1+ doses of pentavalent	-	25.9		
vaccine: 1+ doses of Rota virus vaccine	0.003	18.3		

**Fig 2:**
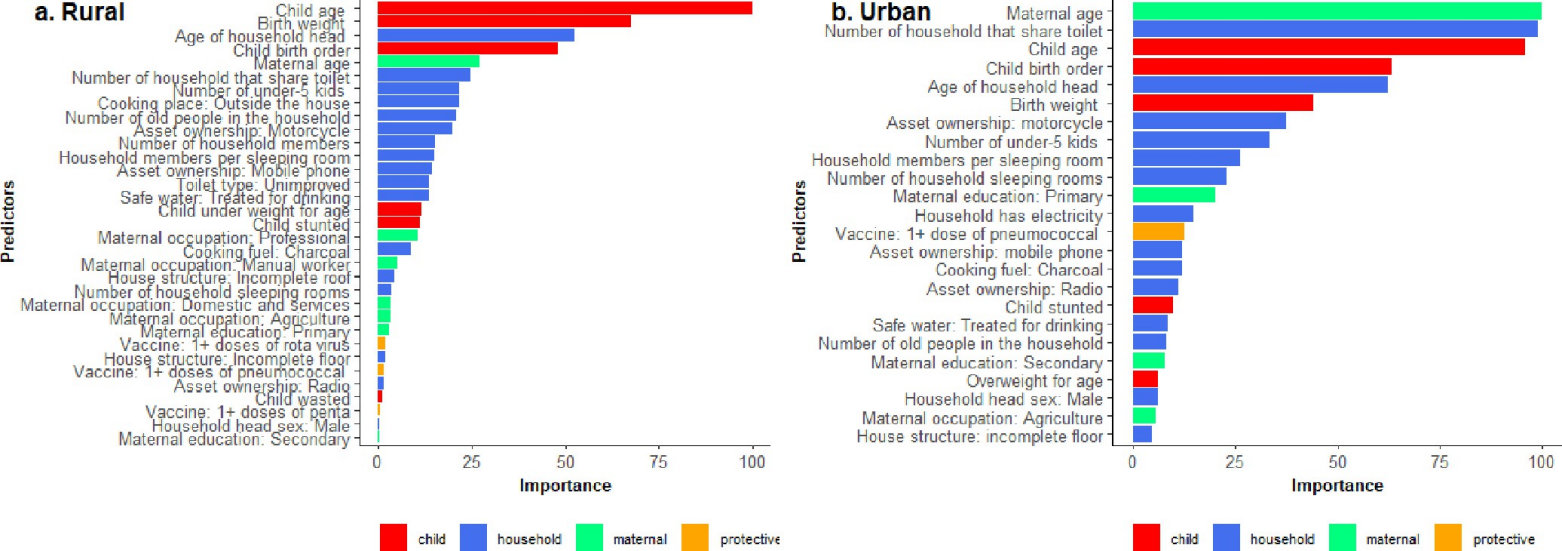
Key household and individual predictors of suspected diarrhoea in rural and urban areas identified through the analysis of 2006–2016 Uganda demographic health surveys data. The identification of variables is based on the best performing ML model, the GBM.

Fig 2 shows the order of importance of the predictors of diarrhoea among rural and urban resident children based on ML (gradient boosted model).

In both places of residence, the individual (child characteristics) predictors were child age, childbirth weight and childbirth order, while nutritional status was among the identified predictors of diarrhoea among urban residents. In rural areas, the traditional logistic model did not identify birth weight as an important predictor, while in urban areas, the traditional logistic model did not consider birth weight, birth order and nutritional status (Table 4). The maternal predictors of suspected child diarrhoea among rural and urban resident children were maternal occupation, age and education (Table 4). Exposure to smoking (indoor and type of fuel used for cooking), hygiene and sanitation measures (number of people sharing a toilet and unimproved toilet), wealth measures (possession of radio, electricity access), household congestion measures (number of household rooms for sleeping and number of people per sleeping room), and household head characteristics (gender and age) were identified as important household predictors under GBM. Furthermore, pentavalent and rotavirus vaccines were also indicated as an important predictor of diarrhoea among rural resident but not for urban residents (Table 4).

### Key household and individual predictors of ARI in rural and urban areas

Table 5 presents the differences in identified predictors of ARI in traditional logistic regression and GBM. The traditional logistic regression and GBM model share the same predictor under maternal characteristics and household characteristics themes; however, some predictors under the child characteristics theme in the GBM were not identified under the traditional logistic model. In addition, the traditional logistics model did not consider all nutritional status variables, including birth weight (Table 5).

**Table 5:** Differences in identified predictors of ARI in traditional logistic regression and GBM.

	Rural		Urban	
	Logistic regression	GBM	Logistic regression	GBM
Variables	P-value	Level of importance	P-value	Level of importance
**Child Characteristics**				
Child age	0.14	100	_	95.8
Childbirth order	0.083	47.9	0.049	63.2
Birth weight	-	67.8	0.088	44.2
Childbirth category: Singleton			0.997	-
Nutritional status: Stunted	-	11	-	9.7
Nutritional status: underweight	-	11.5	-	6.1
Nutritional status: wasted	-	1.3	0.994	-
**Maternal characteristic**				
Maternal age		27		100
Maternal occupation: Manual worker				
Maternal occupation: Professional	-	10.6		
Maternal occupation: Agriculture	0.008	3.3	-	5.8
Maternal occupation: Domestic and services	0.03	3.4	0.993	-
Maternal occupation: Manual worker	0.001	4.9		
Education level: Primary	-	3.1	-	20.3
Maternal education: Secondary	-	0.1	-	7.9
Education level: Higher level	0.982	-	-	-
Marital status: Married	0.151	-	0.179	-
**Household characteristics**				
*Hygiene and sanitation*				
Toilet Type: Unimproved	0.083	13.7		
Number of households that share toilet	-	24.6	0.015	98.8
Household treats water for drinking	0.102	13.5	-	8.7
*Household asset possession*				
Household has electricity	0.086	-	0.012	14.7
Asset ownership: Radio	-	1.5	-	11.1
Asset ownership: Mobile phone	-	14.5	0.005	12.1
Asset ownership: Motorcycle	-	19.7	0.004	37.5
Household asset: TV	0.056	-		
*Household congestion measure*				
Number of household members	0.017	15.4	-	23
Number of household members per room	0.039	15	-	26.1
Number of sleep rooms	0.019	3.7	0.144	-
Number of old people in the household		21	-	8.3
Number of under-5 children	0.054	21.6	0.002	33.5
*Household head characteristics*	-	-		
Age of household head	0.135	52.7	-	62.5
Household head sex: Male	-	0.3	0.079	5.9
*Exposure to smoking*				
Cooking place: Separate room or house	0.014	21.4		
Cooking fuel: Charcoal	0.098	8.7	-	12
*Household structure*				
Incomplete floor	0.163	1.9	-	4.7
Incomplete roof	-	4.4		
**Child vaccination**				
vaccine: 1+ doses of pentavalent vaccine	-	0.6		
vaccine: 1+ doses of pneumococcal vaccine	-	1.5	-	12.8
vaccine: 1+ doses of Rota virus vaccine	0.045	1.9		

**Fig 3:**
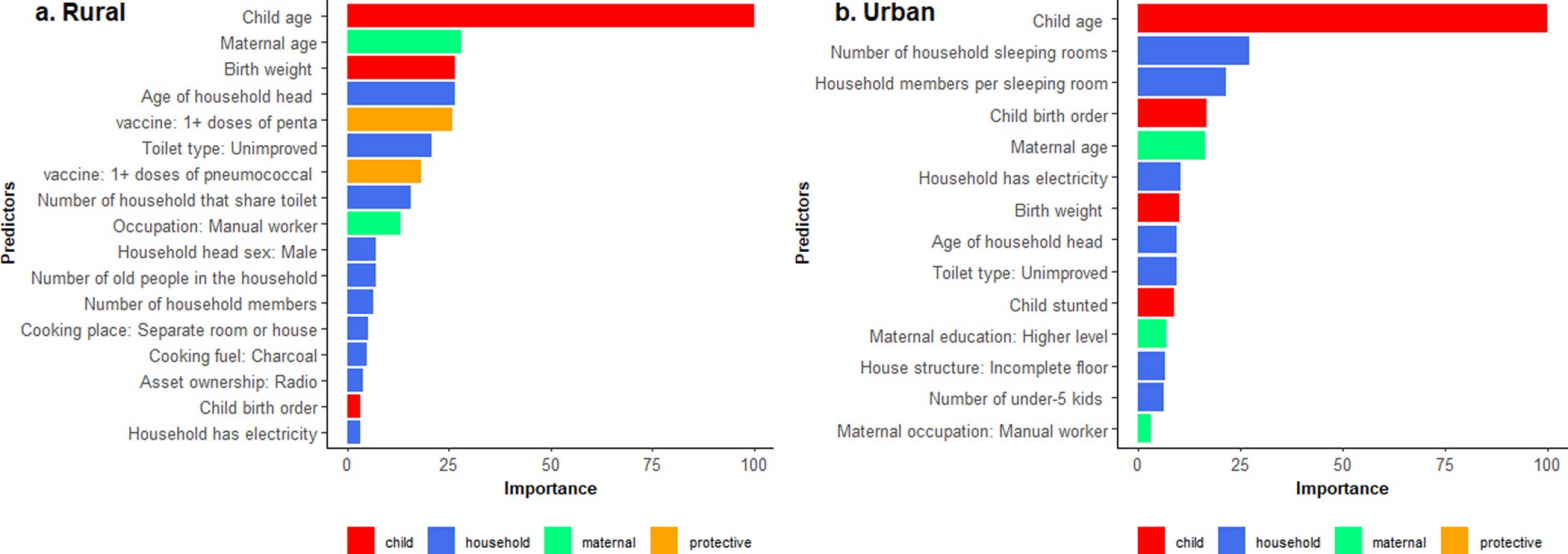
Key household and individual predictors of ARI in rural and urban areas identified through the analysis of 2006–2016 Uganda demographic health surveys data. The identification of variables is based on the best performing ML model, the GBM.

Fig 3 shows the identified predictors of ARI in rural and urban areas.

In rural areas, considering the level of significance (5%) as the criteria for selecting the potential predictors, occupation, treatment of drinking water and all congestion measures, including having a separate room or space for cooking, would be considered as the potential predictors under the traditional logistic model. Under GBM, a range of variables in each category were identified (Table 5). Similarly, based on the level of significance (5%), most of the variables under the child characteristics’ theme, maternal characteristics’ theme, exposure to smoking and being vaccinated may not be considered key predictors under the traditional logistic model in urban areas (Table 5). The exclusion of vaccination variables such as the pentavalent vaccine under the traditional model could be due to the poor performance of traditional models on variables with rare events.

### Association of identified predictors with the occurrence of ARI and diarrhoea

**Fig 4:**
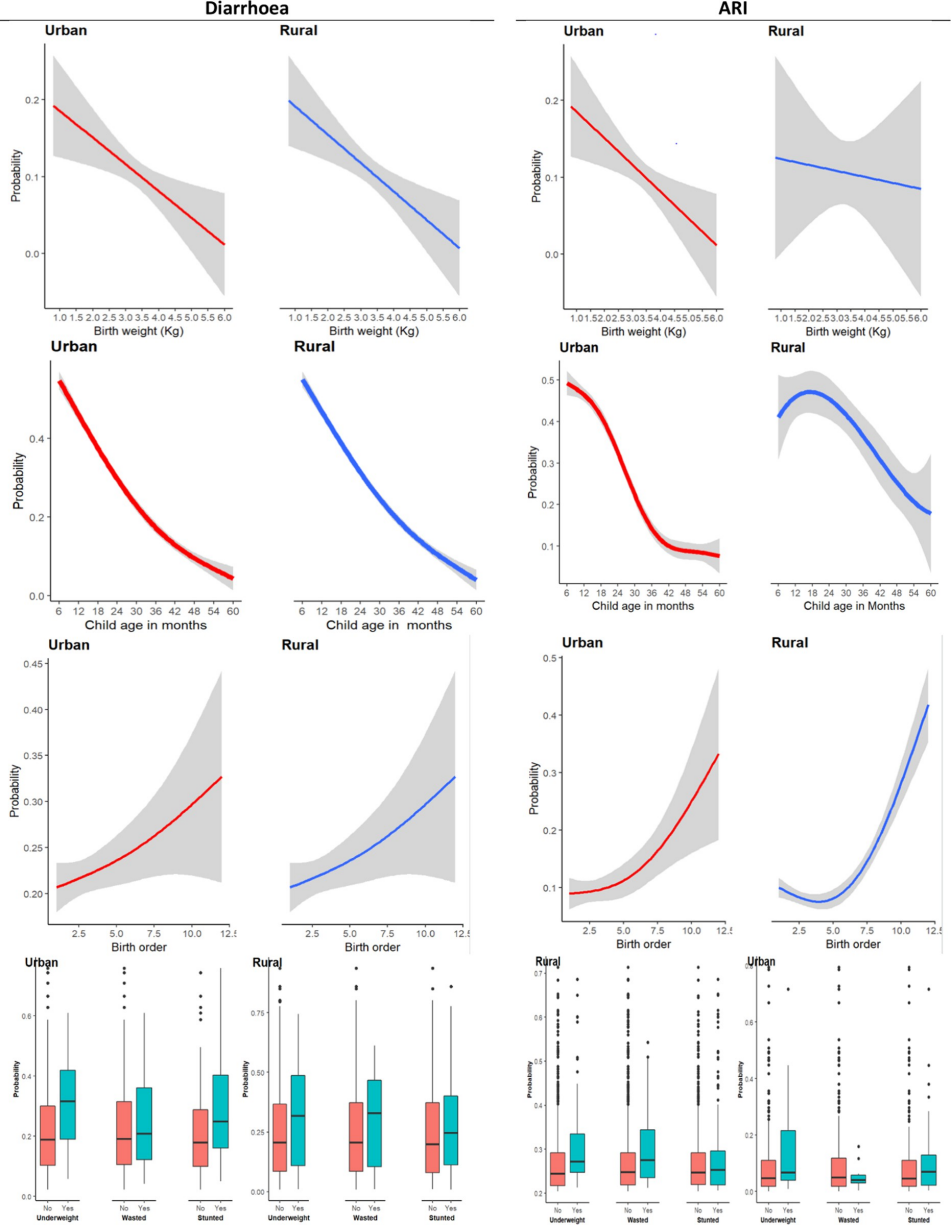
Association between children’s characteristics and child morbidities.

In both places of residence, the association between child age and diarrhoea is observed to be inversely associated with child age (Fig 4). The occurrence appeared to be high among children aged 6–36 months (Fig 4). In both places of residence, birth weight (Fig 4) appears to be almost linearly negatively related to diarrhoea. At the same time, childbirth order is positively related to suspected diarrhoea (Fig 4). All nutritional status measures (underweight, wasted, and stunting) were associated with the high occurrence of suspected diarrhoea (Fig 4).

**Fig 5:**
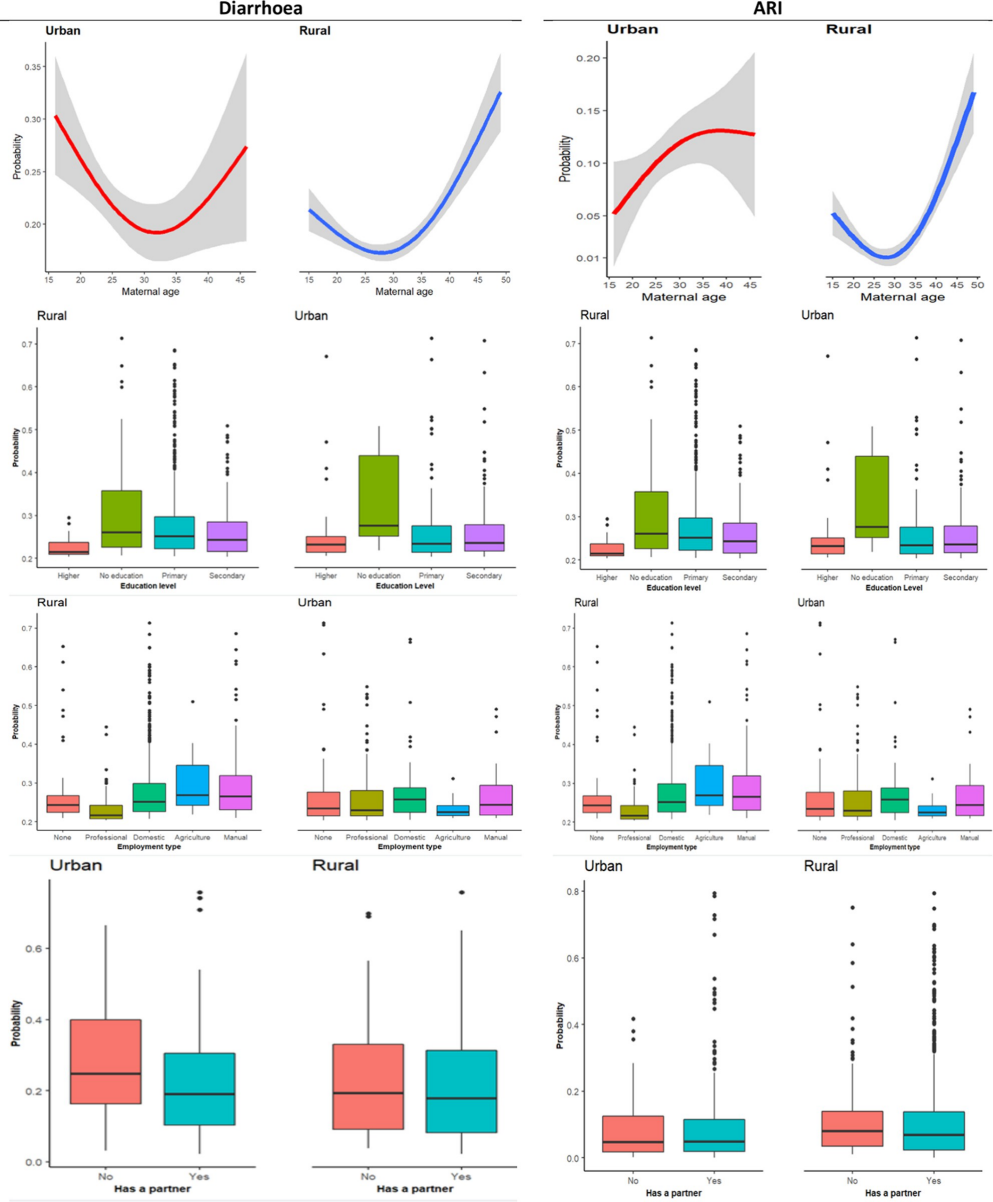
Association between maternal characteristics and child morbidities.

The relationship between diarrhoea and maternal age is non-linear (almost U-shaped curve), with a sharp reduction observed between 15–20 years and a sharp increase observed among those whose mothers were aged 35 years and above (Fig 5). The high likelihood of diarrhoea is also observed among children whose mothers are not educated (Fig 4) and those whose mothers’ occupations are not professional (Fig 5).

**Fig 6:**
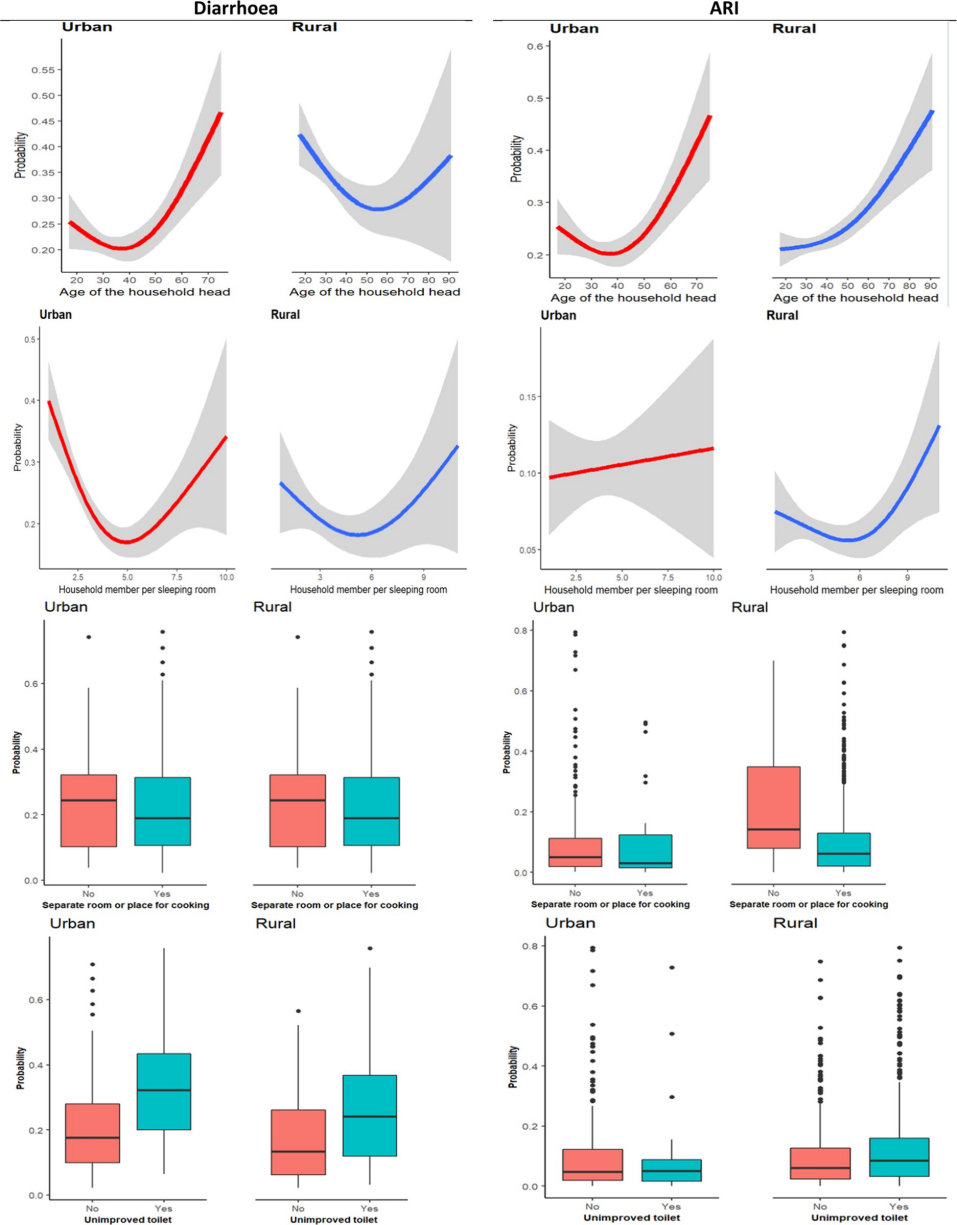
Association of household characteristics with child morbidities.

Additionally, diarrhoea is likely to be high among children whose households had an unimproved toilet and had not a separate place or room for cooking (Fig 6). The relationship between diarrhoea and the household head as well as the number of household members per room, is non-linear (Fig 6).

**Fig 7:**
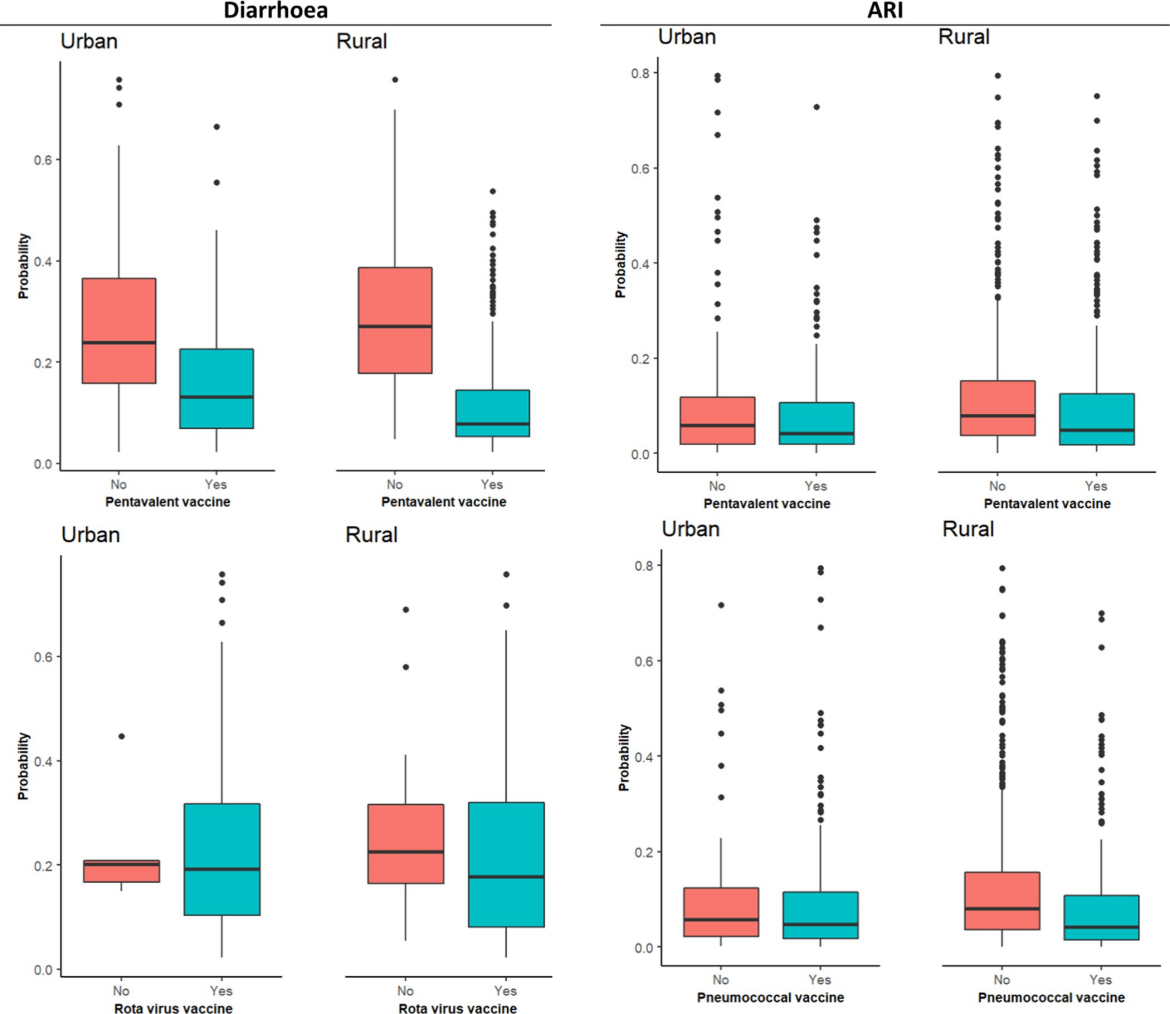
Association of vaccination with child morbidities.

Furthermore, in both places of residence, diarrhoea was high among children who had not received the pentavalent vaccine (Fig 7). A substantial association between the rotavirus vaccine and diarrhoeas is observed among rural residents (Fig 7).

Table 6 shows the differences in identified predictors of ARI and diarrhoea between the places of residence. The analysis shows how diarrhoea and ARI share almost the same predicators, with modest differences. The differences in the identified predictors between places of residence for each morbidity were also modest.

**Table 6:** Differences in identified predictors of pneumonia and diarrhoea between the places of residence.

	Diarrhoea		Pneumonia	
	Urban	Rural	Urban	rural
**Child Characteristics**				
Child age	yes	Yes	yes	yes
Childbirth order	yes	Yes	yes	yes
Birth weight	yes	Yes	yes	yes
Nutritional status: Stunted	yes	No	yes	yes
Nutritional status: underweight	no	No	yes	yes
Nutritional status: wasted	no	no	no	yes
**Maternal characteristic**				
Maternal age	yes	yes	yes	yes
Maternal occupation	yes	yes	yes	yes
Maternal education	yes	no	yes	yes
Marital status: Married	no	no	no	yes
**Household characteristics**				
*Hygiene and sanitation*				
Toilet Type: Unimproved	yes	yes	no	yes
Household treats water for drinking	no	no	yes	yes
Number of households that share toilet	no	yes	yes	yes
*Household asset position*				
Household has electricity	yes	yes	yes	no
Household asset: TV	no	no	no	no
Household asset: radio	no	yes	yes	yes
Asset ownership: Mobile phone	no	no	yes	yes
Asset ownership: Motorcycle	no	no	yes	yes
*Household congestion measure*				
Number of household members	no	yes	yes	yes
Number of household members per room	yes	no	yes	yes
Number of households sleeping rooms	yes	no	yes	yes
Number of under-5 children	yes	no	yes	yes
Number of old people in the household	no	yes	yes	yes
*Household head characteristics*				
Age of household head	yes	yes	yes	yes
Household head sex: Male	no	yes	yes	yes
*Household structure*				
Incomplete floor	yes	no	yes	yes
Incomplete roof	no	no	no	yes
**Child vaccination**				
vaccine: 1+ doses of pentavalent vaccine	no	yes		yes
vaccine: 1+ doses of pneumococcal vaccine	no	yes	yes	yes
vaccine: 1+ rota virus vaccine	no	no	no	yes
*Exposure to smoking*				
Cooking place: Separate room or house	no	no	no	yes
Cooking fuel: Charcoal	no	no	yes	yes

## Discussion

This is the first study that has applied ML to cross-sectional population health data to identify the key predictors of ARI and diarrhoea among the under-five year of age in a low-income setting. The study has demonstrated how household, maternal, individual characteristics as well as information on protective interventions obtained through cross-sectional studies could be used to predict the occurrence of health outcomes, including health behaviours. Thus, this study contributes to the knowledge of the application of ML techniques in population health and social science research. The analysis approach may be replicated in other studies to develop prediction models. Additionally, the study contributes to the understanding of the variation in the effect of predictors across the places of residence. It explains the trends in the prevalence of diarrhoea and ARI in Uganda and how the identified predictors could contribute to the design of interventions.

Findings from this work point out two important points. First, for the last decade (2006–2016), the study’s results show slow progress in reducing the prevalence of diarrhoea and ARI. Such slow progress in a decade demonstrates how Uganda is lagging in achieving the integrated global action plan for ending pneumonia and diarrhoea by 2025 [66]. Further, the slow progress demonstrated the need to rethink strategies to help the country achieve the SDG objective of reducing deaths due to preventable causes. Understanding the context-specific morbidities’ predictors contributes to identifying and implementing area-specific interventions that may accelerate progress. Second, the ML in the form gradient boosted model (GBM) was the best ML model in generating the predictors of ARI and diarrhoea. For both testing and training datasets, the identified predictors of ARI in urban and rural areas under GBM had an accuracy of 100% in predictions, indicating that the model could correctly predict those with and without the diseases. Similarly, for both testing and training datasets, the identified predictors of suspected diarrhoea in urban and rural areas under GBM had a prediction accuracy of 100% and close to 70%, respectively. Therefore, the interpretation of the results and conclusions are based on the GBM predictions.

Based on GBM, I identified moderate differences in the predictors of ARI and diarrhoea between rural and urban residents. The identified key predictors were categorised as child, maternal and household characteristics, as well as protective interventions. While most of the predictors in the traditional logistic regression model appeared in the GBM, a substantial number of predictors in the GBM did not appear in the traditional logistic model. Some of the variables may not be considered due to the selection of the variables on the level of significance (p-value). For instance, child nutritional status, hygiene and sanitation measures, asset position, household health characteristics and household structure were not identified as key predictors of suspected diarrhoea and pneumonia. Based on GBM predictions, I discuss the association of identified predictors in each category with the occurrence of diarrhoea and pneumonia in the subsequent subsections.

### Individual characteristics and child morbidity

Under the individual characteristics theme, the identified important predictors were child age, birth weight, birth order, and nutrition status. The findings affirm a non-linear relationship between children’s age and the occurrence of ARI and diarrhoea as has been indicated in another study in similar settings [41]. An inverse relationship between morbidities’ prevalence and children’s age was observed and it appeared to be high among children aged 6–36 months. The high prevalence of the diseases in the early months of life indicates children’s exposure to morbidities’ risk factors such as poor nutritional status, poor sanitation, and hygiene that this study identified as key predictors. This finding provides an insight into the need for interventions that target children within the first 36 months of life. Indeed some studies have indicated the need for targeting the first 1000 days of life with health and well-being interventions [67, 68].

Further, the study results show a linear relationship between children’s birth weight and the occurrence of ARI and diarrhoea. In both places of residence, the high occurrence of ARI and diarrhoea was observed among children born with a birth weight of less than 2500 grams. The association of LBW with diarrhoea and ARI has been indicated in some other studies [48, 50–52]. The thinking could be that LBW is usually mediated by some determinants of the prevalence of ARI and diarrhoea, such as being underweight for age and anaemia, whose risks are high among children born as LBW. The association of LBW with other child risk factors such as underweight and anaemia has been indicated in several studies [45–48]. This finding suggests community interventions that target mothers or families with extreme birth weight (LBW) children, in addition to LBW preventive interventions. Exclusive breastfeeding as a recommended intervention for new-born [69] has been indicated to lower the probability of pneumonia deaths among LBW [70], while prenatal interventions focusing on better nutrition and lifestyle as well as screening for pregnancy danger signs such as diabetes and infections have been indicated as key interventions for controlling LBW [71].

Furthermore, a positive linear relationship between birth order and morbidities was observed in both places of residence, which has been reported in other studies done in developing countries [72–74]. The relationship between birth order and child morbidity could result from the possible correlation between the number of children and birth positions. Morosow and Kolk, 2020 argue that one of the reasons why earlier-born siblings cope better is that parents’ resources (including non-economic resources such as time) are usually fixed, and consequently, having more children may lead to fewer available resources per child [75]. The increase in the number of siblings spacing between them dilutes the time, and material resources parents can give to each child [76, 77]. Ultimately, the dilution of resources inhibits the well-being of later birth orders. For instance, the lower birth order, particularly the firstborns, may receive better attention from the parents than those of later birth order or born later [77].

Finally, poor nutrition statuses were associated with a high likelihood of child morbidities. The relationship between poor nutritional status and child morbidities has been well documented in studies done in some countries in Africa and Asia [78–80]. On the one hand, poor nutrition could result from exposure to poor nutrition during preconception and while pregnant, which usually leads to LBW. Good nutrition in the preconception period is crucial to ensure that women have enough nutrient stores to support both fetal and maternal nutrition throughout gestation [81]. On the other hand, poor nutrition could be due to poor maternal and feeding, hygiene, and sanitation [81]. Such an argument showcases the need for a life course approach in delivering nutrition interventions. Notably, efforts could be focused on improving intervention coverage focusing on the most vulnerable.

### Maternal characteristics and child morbidity

The identified predictors under the maternal characteristics theme were marital status, maternal education, maternal age, and maternal occupation, which were associated with the likelihood of child morbidities. The relationship between maternal age and the occurrence was non-linear, with a high likelihood observed among children whose mothers were aged less than 20 years and 35 years plus. The association between child health and the caretakers’ age results from caretaker’s age on their ability and autonomy in child-rearing [82]. Adolescent age could also be associated with education levels, occupation, and marital status, which this study identified as key predictors. Similarly, maternal age could be correlated with the number of children. Therefore, the high likelihood of morbidities among children aged 35 years plus could be related to the high number of children or increasing birth order, identified in this study as one of the key predictors.

Furthermore, the higher the maternal education and professional occupation, the higher the likelihood of child morbidity. This is expected as education and professional (elite) occupations are correlated. Evidence has indicated how educated mothers and those engaged in professional work may have better knowledge and resources of child-rearing. Such findings suggest childcare interventions that target uneducated and younger women. Such information and learning from the COVID-19 experience could perhaps be disseminated through all media platforms, including social media [83], social health campaigns and integration of the sensitisation messages within community and facility health workers’ routine work.

### Household characteristics and child morbidity

Household head age, asset ownership, type of the toilet, availability of a separate room or place for cooking, and household members were among the predictors of child morbidities. Important to note is that the identified household characteristics measure household wealth positions and resources access [84–86], which affect the health and well-being of household members, including children. The findings show a non-linear relationship between the age of the household’s heads and the occurrence of child morbidities. Just like maternal age, the likelihood of morbidities appeared to be high among children of adolescent household head families and those aged beyond 50 years of age. Furthermore, consistent with other studies [72–74], the likelihood of morbidities was observed among children living in households with unimproved toilets and households without a separate room or place for cooking. Improving households’ ability to address household-based risk factors that affect children’s health may benefit from implementing interventions that are responsive to the unique structure and composition of the household.

### Protective interventions and child morbidity

Pentavalent, rotavirus, and pneumococcal vaccines were identified among important predictors of ARI and diarrhoea among rural resident children. In contrast, pneumococcal was identified among the key predictors of ARI in urban resident children. In addition, the effect of other immunisation vaccines, such as measles, on reducing the occurrence of diarrhoea and pneumonia has been reported in some studies [4, 87–89].

### Strengths and limitations

This study, which deliberately focused on identifying the community and household predictors of ARI and diarrhoea, makes two major contributions. First, to the best of my knowledge, the study is the first to apply an algorithmic modelling approach to identify household and individual predictors of pneumonia and diarrhoea in LMICs settings based on cross-sectional surveys. Hence, the study contributes to the knowledge of the application of ML techniques in population health and social science research. Second, the study contributes to understanding risk factors and determinants of ARI and diarrhoea in urban and rural settings, which could be used to measure vulnerable groups in each place of residence.

The limitation of DHS relates to the nature of cross-sectional studies on retrospectively collecting information on human behaviours and events. For instance, the occurrences of diarrhoea and measures of ARI and birth weight in the DHS rely on women’s ability to recall. However, the DHS consideration of children born in the last five years preceding the survey may minimise such bias. Further, the DHS consideration of collecting data on occurrences of ARI and diarrhoea episodes and symptoms that occurred in the two weeks preceding the day of the interview may also minimise the reporting bias of the morbidities’ symptoms and occurrences. Additionally, the reporting of the occurrences of diarrhoea and ARI are subject to the symptoms and thus may not provide accurate information as it would have been if clinical notes were available [90, 91]. However, for ARI, the questions on if the child had short, rapid breathing, which was chest-related or difficult breathing, are asked as a measure of the occurrence of ARI, which may increase the accuracy in the estimation of ARI prevalence.

Further, the analysis of data in this study considered children who were present at home during the interview could have reduced the reporting bias (under-reporting). One of the reasons is that the parents may not know the health status of the children that are not living with them at home.

Additionally, the ML approach to data analysis does not mean that there are no other variables in the dataset associated with suspected diarrhoea and ARI, in addition to including only variables that account for the most variance in the study outcomes. Furthermore, the study’s findings may be limited to Uganda’s context; however, these may apply in other countries with similar contexts. Particularly, the study unveils the need for using algorithmic modelling approaches to identify a set of vulnerable groups in cross-sectional surveys.

Finally, the use of ML leads to interpretation challenges [63], in particular, the causal-effect interpretation may be challenging since the selection of important variables is based on the extrapolation of patterns found in the labelled training data [92]. Nevertheless, I explain the mechanisms through which the identified topmost variables are associated with the study outcomes in relation to the available literature.

## Conclusion

The descriptive statistics on trends in the prevalence of ARI and diarrhoea show how Uganda has progressed slowly in reducing ARI and diarrhoea in a decade (2006–2016). Such progress raises worries if, at the same pace, the country will be able to achieve an integrated global action plan for ending pneumonia and diarrhoea by 2025 and SDG targets related to reducing deaths due to preventable causes. To accelerate progress, I argue that health interventions could address the study’s identified diarrhoea and ARI predictor. Using ML analysis techniques, I identified a set of variables some of which would not appear when using the traditional logistic model (e.g.: household structure and composition, birth weight and child nutritional status), which shows how the approach may contribute to the design of holistic interventions. Alongside traditional models, ML could be applied in generating research hypotheses and providing insight into the selection of key variables that should be considered in the model.

The findings confirm the notion that ARI and diarrhoea risk factors and determinants overlap. These factors relate to the household’s structure and composition characterised by poor hygiene and sanitation, poor household environments; maternal socio-economic factors such as education, occupation, and fertility (birth order); and individual risk factors such as child age and birth weight and nutritional status. Furthermore, all the identified factors appear to be correlated and can be addressed with the same interventions. Additionally, while the predictors were different in the order of importance, they appeared to be the same in both urban and rural areas. Such evidence indicates that similar interventions may benefit rural and urban residents within the identified risk factors categories.

Furthermore, these results underscore the need for the life course and multisectoral approaches in addressing some of the identified interventions. First, the association between LBW, younger maternal age and child’s birth order confirms the need for life-course interventions that improve children’s health and well-being, including their mothers from pregnancy conception and early years of age [93, 94]. For instance, early pregnancy interventions such as good nutrition and identification of other risk factors for extreme small birth weight (such as infections) are meant to address the factors that contribute to the LBW during the foetus stage of child development [95]. We also know how LBW children are associated with a high likelihood of poor nutritional status and other morbidities, including ARI and diarrhoea. Therefore, immediately after delivery (day 0) up to two years of age and beyond, special health and social interventions could target LBW children, including younger mothers as well as households headed by younger and older caretakers. Secondly, for optimal implementation of interventions that address some of the identified predictors or factors such as nutrition status, immunisation, hygiene, and sanitation, it is important to leverage a multisectoral approach for collaboration and integration. Learning from COVID-19 response within and outside Uganda, addressing these challenges requires responsible sectors working towards integrated services. Finally, some of the factors such as congestion, household structure, household composition, asset ownership, access to electricity, in-door pollution, unimproved toilet, unemployment and informal employment, and lower education levels are measures of deprived individuals and communities such as slums in urban settings; and these were same in rural and urban areas. These findings underscore the importance of reaching all rural and urban residents with the same intervention package. However, the mode of delivery may differ given the differences in residential complexity. Beyond these findings, I recommend a study on understanding how these measures could be used to generate the deprivation index and how health outcomes are distributed across the different groups.

## Supporting information

S1 Fig Percentage of under-five aged children with symptoms of acute respiratory infections and diarrhoea in sub-Saharan countries with recent demographic health surveys (2013–2018) as of August 2020. (TIF)

S1 Table Variables for each of the categories based on 2006–2016 Uganda demographic health surveys. (DOCX)

S2 Table Maternal, household and child characteristics. (DOCX)
