# Supplementary figures and images for: Machine learning predictive modelling for identification of predictors of acute respiratory infection and diarrhoea in Uganda’s rural and urban settings
Source: PLOS Glob Public Health. 2022 May 11;2(5):e0000430. doi: 10.1371/journal.pgph.0000430 (PMC10021828; doi:10.1371/journal.pgph.0000430)

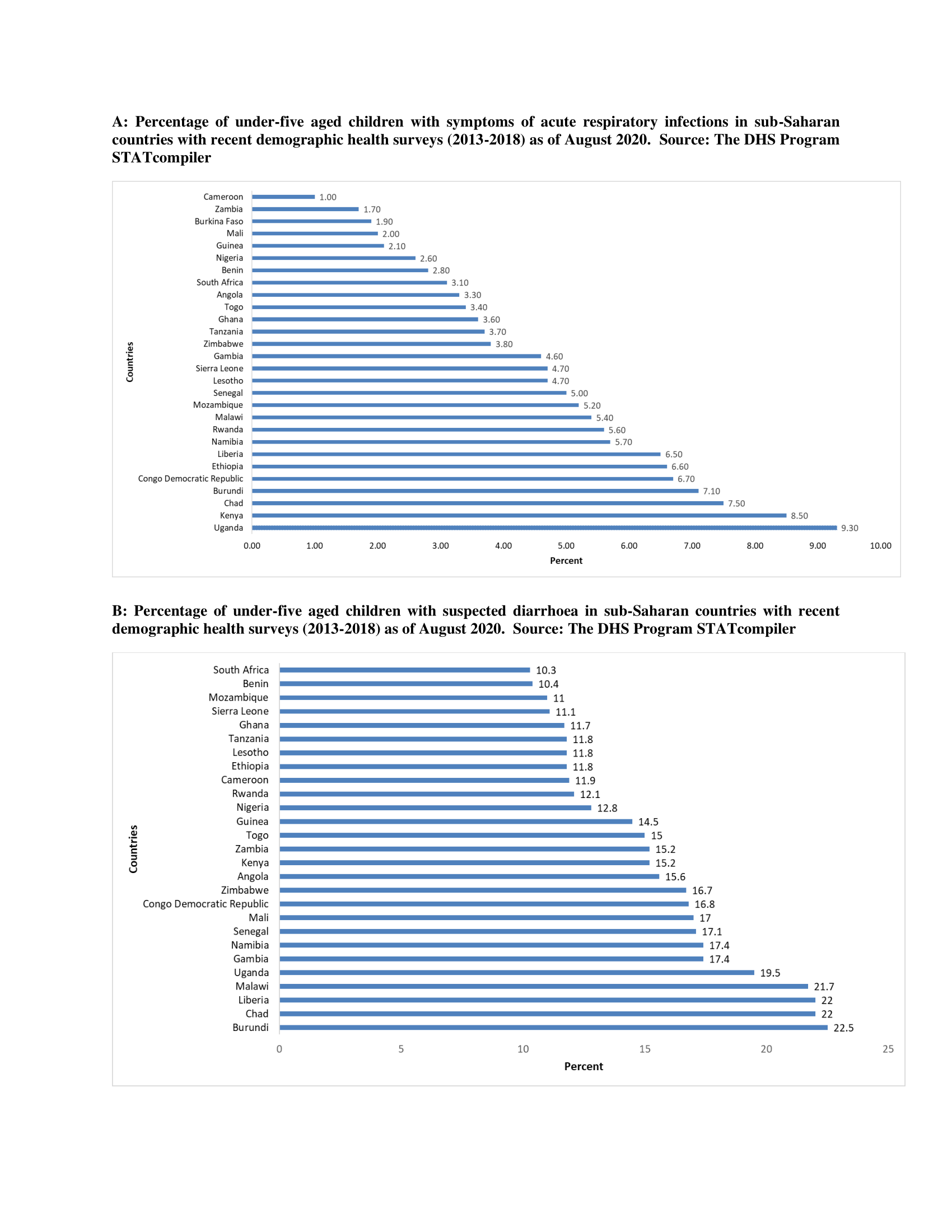

Supplement: S1 Fig — (TIF) [file pgph.0000430.s001.tif]
