# Supplementary material for: Machine learning predictive modelling for identification of predictors of acute respiratory infection and diarrhoea in Uganda’s rural and urban settings
Source: PLOS Glob Public Health. 2022 May 11;2(5):e0000430. doi: 10.1371/journal.pgph.0000430 (PMC10021828; doi:10.1371/journal.pgph.0000430)
Supplement: S1 Table — (DOCX) [file pgph.0000430.s002.docx]

**S1 Table: Variables for each of the categories based on 2006-2016 Uganda demographic health surveys**

|  | **Variables** | **Data type and categorisation** |
| --- | --- | --- |
|  | **Outcome categorisation** | |
| 1 | Had ARI | The measurement of ARI is based on the occurrence of short, rapid breathing that is chest-related and/or difficult breathing that is chest-related that is based on women ability to recall. This was categorised as 1 – for the presence and 0 – otherwise. |
| 2 | Had diarrhoea | Women are asked to recall if their children have had diarrhoea in the last 2 weeks. This was categorised as 1 – for the presence and 0 – otherwise. |
|  | **Set of predictors** | |
|  | *Mothers’ demographic position* |  |
| 1 | Mothers age | Continuous variable |
| 2 | Household headed by a female | Dummy variable that was coded as 0 – male-headed and 1 – female-headed households |
| 3 | Mother’s education | Ordinal variable that was coded as 0 – no education level at all 1 – primary education level 2 – secondary education level and 3 – tertiary education level |
| 4 | Mother’s occupations | Nominal variable that was categorised as 0 – no occupation at all, 1 – professional and business work, 2 – agriculture and self-employment, 3 – domestic work, 4 – manual work |
| 6 | Marital status | Dummy variable that was coded as 0 – not married or staying with the partner and 1 – married or staying with a partner. The widows divorced or separated were considered as 0, while married or staying with the partner as if married were considered as 1. |
| *2.0.* | *Household assets* |  |
| 7 | Car | These were all considered as dummy variables coded as 0 – household without the specified asset and 1 – household with the specified asset. The missing and don’t know were considered as 0. |
| 8 | Motorcycle |  |
| 9 | Fridge |  |
| 10 | Radio and TV |  |
| 11 | Electricity availability |  |
| *3.0.* | *Household Environmental characteristics* |  |
| 12 | Number of people in a house | Continuous variable. Missing fields were considered as 0 |
| 13 | Number of children people in a house | Continuous variable. Missing fields were considered as 0 |
| 14 | Number of old people in a house | Continuous variable. Missing fields were considered as 0 |
| 15 | House’s wall structure | Dummy variable that was coded as 0 – incomplete and 1 – complete. Complete is considered for rudimentary and modern building materials. |
| 16 | House’s roof structure | Dummy variable that was categorised as 0 – incomplete and 1 – complete. Complete is considered for rudimentary and modern building materials. |
| 17 | House floor structure | Dummy variable that was coded as 0 – incomplete and 1 – complete. Complete is considered for rudimentary and modern building materials. |
| 18 | Cooking | Nominal variable that was categorised as 1 – Electricity, 2 – LPG/Gas, 3 – wood, wood, straw/shrub/grass, crops, and animal dung, and 4 – charcoal |
| 19 | Cooking done outside the house | Dummy variable that was categorised as 1 – household cooking is done in a separate house or room, 0 – otherwise |
| 20 | Toilet shared | Coded as 0 – toilets are not shared with other households 1 – otherwise |
| 21 | Drinking water treated | Dummy variable categorised as 0 – household drinking water is not treated 1 – otherwise |
| 22 | Protected water sources | Dummy variable categorised as 0 – household has access to protected water 1 – otherwise |
| 23 | Improved toilet | Dummy variable categorised as 0 – household has access to improved toilet 1 – otherwise |
| *4.0.* | *Child characteristics* |  |
| 24 | Child sex | Dummy variable coded as 0 – male and 1 – female |
| 25 | Child age | Continuous variable measured in months |
| 26 | Birth weight | Continuous variable measured in grams |
| 27 | Weight for age | Dummy variable coded as 0 – for the standardized (standard deviation) weight for age <-2 (below the World Health Organization mean child growth standard) and 1 – otherwise |
| 28 | Weight for height | Dummy variable that was coded as 0 – for the standardized (standard deviation) weight for height <-2 and 1 – otherwise |
| 29 | Height for age | Dummy variable that was categorised as 0 – for standardized (standard deviation) height for age <-2 (below the World Health Organization mean child growth standard) and 1 – otherwise |
| 5.0 | Vaccination |  |
| 30 | Pentavalent vaccine | Dummy variable that was coded as 1 – if at least one recommended doses were received and 0 – otherwise |
| 31 | Rota virus vaccine |  |
| 32 | Pneumococcal vaccine |  |
