# Supplementary material for: Machine learning predictive modelling for identification of predictors of acute respiratory infection and diarrhoea in Uganda’s rural and urban settings
Source: PLOS Glob Public Health. 2022 May 11;2(5):e0000430. doi: 10.1371/journal.pgph.0000430 (PMC10021828; doi:10.1371/journal.pgph.0000430)
Supplement: S2 Table — (DOCX) [file pgph.0000430.s003.docx]

**S2 Table 1: Maternal and household characteristics**

|  | **2006** | | **2011** | | **2016** | |
| --- | --- | --- | --- | --- | --- | --- |
|  | Weighted sample | % | Weighted sample | % | Weighted sample | % |
| **Education level** |  |  |  |  |  |  |
| No education | 1,646 | 22.7 | 1036 | 14.7 | 1501 | 11.2 |
| Primary | 4,609 | 63.6 | 4489 | 63.6 | 8214 | 61.5 |
| Secondary | 818 | 11.3 | 1277 | 18.1 | 2777 | 20.8 |
| Higher | 175 | 2.4 | 252 | 3.6 | 871 | 6.5 |
| **Occupation** |  |  |  |  |  |  |
| No occupation at all | 564 | 7.8 | 1,478 | 21.0 | 2328 | 17.4 |
| Professional and business work | 687 | 9.5 | 275 | 3.9 | 2221 | 16.6 |
| Agriculture and self-employment | 5,472 | 75.5 | 4,064 | 57.6 | 6192 | 46.3 |
| Domestic work | 191 | 2.6 | 1,237 | 17.54 | 626 | 4.7 |
| Manual work | 335 | 4.6 | - | - | 1996 | 14.9 |
| **Household owns a TV** |  |  |  |  |  |  |
| No | 6,908 | 95.3 | 6348 | 90.0 | 11329 | 84.8 |
| Yes | 341 | 4.7 | 707 | 10.0 | 2035 | 15.2 |
| **Household owns Radio** |  |  |  |  |  |  |
| No | 2,770 | 38.2 | 2400 | 34.0 | 5715 | 42.8 |
| Yes | 4,479 | 61.8 | 4655 | 66.0 | 7649 | 57.2 |
| **Household treats water for drinking** |  |  |  |  |  |  |
| No | 4,684 | 64.6 | 4173 | 59.2 | 6563 | 49.1 |
| Yes | 2,564 | 35.4 | 2882 | 40.9 | 6801 | 50.9 |
| **Household toilet shared with other households** |  |  |  |  |  |  |
| No | 4,435 | 61.2 | 4599 | 65.2 | 8537 | 63.9 |
| Yes | 2,814 | 38.8 | 2455 | 34.8 | 4827 | 36.1 |
| **Household place of cooking** |  |  |  |  |  |  |
| Inside the house | 838 | 11.6 | 575 | 8.2 | 1121 | 8.4 |
| Outside the house | 6,411 | 88.4 | 6479 | 91.8 | 12243 | 91.6 |
| **Main cooking fuel** |  |  |  |  |  |  |
| Electricity | 3 | 0.1 | 4 | 0.1 | 6 | 0.0 |
| LPG-gas | 6 | 0.1 | 28 | 0.4 | 50 | 0.4 |
| Wood | 6,349 | 87.6 | 5699 | 80.8 | 10292 | 77.0 |
| Charcoal | 890 | 12.3 | 1324 | 18.8 | 3016 | 22.6 |
| **Roof structure** |  |  |  |  |  |  |
| Incomplete | 3,398 | 46.9 | 2675 | 37.9 | 4285 | 32.1 |
| Complete | 3,850 | 53.1 | 4380 | 62.1 | 9079 | 67.9 |
| **Floor structure** |  |  |  |  |  |  |
| Incomplete | 6,061 | 83.6 | 5340 | 75.7 | 9017 | 67.5 |
| Complete | 1,187 | 16.4 | 1715 | 24.3 | 4347 | 32.5 |
| **Wall structure** |  |  |  |  |  |  |
| Incomplete | 45 | 0.6 | 44 | 0.6 | 1171 | 8.8 |
| Complete | 7,204 | 99.4 | 7011 | 99.4 | 12192 | 91.2 |
| **Marital status** |  |  |  |  |  |  |
| No partner | 904 | 12.5 | 812 | 11.5 | 1874 | 14.0 |
| Has a partner or married | 6,345 | 87.5 | 6243 | 88.5 | 11490 | 86.0 |
| **Sex of the household head** |  |  |  |  |  |  |
| Female | 5,704 | 78.7 | 5634 | 79.9 | 10168 | 76.1 |
| Male | 1,544 | 21.3 | 1420 | 20.1 | 3196 | 23.9 |
| **Car ownership** |  |  |  |  |  |  |
| No | 7,163 | 98.8 | 6,874 | 97.4 | 12,866 | 96.3 |
| Yes | 86 | 1.2 | 180 | 2.6 | 498 | 3.7 |
| **Motorcycle ownership** |  |  |  |  |  |  |
| No | 6,836 | 94.3 | 6,318 | 89.6 | 11,371 | 85.1 |
| Yes | 205 | 2.8 | 631 | 8.9 | 1,646 | 12.3 |
| Non-resident | 208 | 2.9 | 106 | 1.5 | 347 | 2.6 |
| **Maternal age** | Mean =28.9, ±6.8 | | Mean =28.8, ±6.8 | | Mean =28.6, ±6.8 | |
| **Age of the household head** | Mean =36.9, ±11.5 | | Mean =37.3, ±11.7 | | Mean =37.4, ±12.0 | |
| **Number of rooms used for sleeping** | Mean=4.1, ±1.9 | | Mean=4.1, ±1.9 | | Mean=3.6, ±1.7 | |
| **Number of household members** | Mean=6.2, ±2.8 | | Mean=6.6, ±2.6 | | Mean=6.2, ±2.7 | |
| ***Note:*** *The estimates are based on the live children who were staying with the parents or caretakers at the time of interview.* | | | | | | |

**S2 Table 2: Child-related individual characteristics**

|  | **2006** | | **2011** | | **2016** | |
| --- | --- | --- | --- | --- | --- | --- |
|  | Weighted sample | % | Weighted sample | % | Weighted sample | % |
| **Childbirth position** |  |  |  |  |  |  |
| 1 | 1,154 | 15.9 | 1,133 | 16.1 | 2,672 | 20.0 |
| 2 | 1,058 | 14.6 | 1,183 | 16.8 | 2,440 | 18.3 |
| 3 | 1,009 | 13.9 | 1,037 | 14.7 | 2,100 | 15.7 |
| 4 | 913 | 12.6 | 891 | 12.6 | 1,648 | 12.3 |
| 5 | 815 | 11.3 | 733 | 10.4 | 1,328 | 9.9 |
| 6 | 708 | 9.8 | 652 | 9.2 | 967 | 7.2 |
| 7 | 544 | 7.5 | 523 | 7.4 | 799 | 6.0 |
| 8 | 415 | 5.7 | 362 | 5.1 | 565 | 4.2 |
| 9 | 288 | 4.0 | 236 | 3.4 | 383 | 2.9 |
| 10+ | 346 | 4.8 | 304 | 4.4 | 462 | 3.5 |
| **Birth category** |  |  |  |  |  |  |
| Multiple | 196 | 2.7 | 203 | 2.9 | 379 | 2.8 |
| Singleton | 7,052 | 97.3 | 6852 | 97.1 | 12985 | 97.2 |
| **Child sex** |  |  |  |  |  |  |
| Female | 3,555 | 49.0 | 3518 | 49.9 | 6674 | 49.9 |
| Male | 3,693 | 51.0 | 3537 | 50.1 | 6690 | 50.1 |
| **SD height for age<-2** |  |  |  |  |  |  |
| Normal (>=-2) | 1500 | 62.7 | 1,382 | 66.8 | 3,112 | 71.9 |
| Stunted | 891 | 37.3 | 686 | 33.2 | 1,216 | 28.1 |
| **SD weight for height<-2** |  |  |  |  |  |  |
| Normal (>=-2) | 2233 | 93.4 | 1,965 | 95.0 | 4,155 | 96.2 |
| Wasted | 158 | 6.6 | 103 | 5.0 | 166 | 3.8 |
| **SD weight for age<-2** |  |  |  |  |  |  |
| Normal (>=-2) | 2016 | 84.3 | 1,786 | 86.4 | 3,902 | 89.7 |
| Underweight | 376 | 15.7 | 282 | 13.7 | 447 | 10.3 |
| **Received at least one dose of pentavalent vaccine **** |  |  |  |  |  |  |
| No | 3192 | 49.5 | 399 | 6.4 | 5343 | 44.6% |
| Yes | 3258 | 50.5 | 5856 | 93.0 | 6631 | 55.4% |
| **Received at least one dose of rota vaccine **** |  |  |  |  |  |  |
| No | - | - | - | - | 11302 | 94.4 |
| Yes | - | - | - | - | 672 | 5.6 |
| **Received at least one dose of pneumococcal vaccine **** |  |  |  |  |  |  |
| No | - | - | - | - | 7322 | 61.2 |
| Yes | - | - | - | - | 4653 | 38.9 |
| **Birth weight** | Mean =3379.2, ±918.6 | | Mean =3407.3, ±955.8 | | Mean =3341.3, ±823.6 | |
| ***Note:*** *The estimates are based on the live children who were staying with the parents or caretakers at the time of interview.*  *** considered only children aged 6-59 months* | | | | | | |
